# Business-as-usual trends will largely miss 2030 global conservation targets

**DOI:** 10.1007/s13280-024-02085-6

**Published:** 2024-11-07

**Authors:** Ignacio Palomo, Alberto González-García, Paul J. Ferraro, Roldan Muradian, Unai Pascual, Manuel Arboledas, James M. Bullock, Enora Bruley, Erik Gómez-Baggethun, Sandra Lavorel

**Affiliations:** 1 Univ. Grenoble-Alpes, IRD, CNRS, Grenoble INP, INRAE, IGE, 38000 Grenoble, France; 2 Carey Business School and Department of Environmental Health and Engineering, Johns Hopkins University, Baltimore, MD USA; 3 Faculty of Economics, Universidade Federal Fluminense, Niterói, Rio de Janeiro Brazil; 4 Centre for Environment and Development, University of Bern, 3012 Bern, Switzerland; 5 Basque Centre for Climate Change, BC3, 48940 Leioa, Bizkaia Spain; 6 Ikerbasque, Basque Foundation for Science, 48009 Bilbao, Bizkaia Spain; 7 Universidad de Jaén, Jaén, Spain; 8 UK Centre for Ecology & Hydrology (UKCEH), Wallingford, UK; 9 Department of International Environment and Development Studies (Noragric), Faculty of Landscape and Society, Norwegian University of Life Sciences (NMBU), PO Box 5003, 1432 Ås, Norway; 10 Norwegian Institute for Nature Research (NINA), Gaustadalléen 21, 0349 Oslo, Norway; 11 Laboratoire d’Ecologie Alpine, UMR 5553, CNRS–UGA-USMB, CS 40700, 38058 Grenoble Cedex 9, France

**Keywords:** Biodiversity, Deforestation, Environmental targets, Glasgow Declaration, Kunming-Montreal Global Biodiversity Framework, Restoration

## Abstract

To address climate change and global biodiversity loss, the world must hit three important international conservation targets by 2030: protect 30% of terrestrial and marine areas, halt and reverse forest loss, and restore 350 Mha of degraded and deforested landscapes. Here, we (1) provide estimates of the gaps between these globally agreed targets and business-as-usual trends; (2) identify examples of rapid past trend-shifts towards achieving the targets; and (3) link these past trend-shifts to different levers. Our results suggest that under a business-as-usual scenario, the world will fail to achieve all three targets. However, trend-shifts that rapidly “bend the curve” have happened in the past and these should therefore be fostered. These trend-shifts are linked to transformative change levers that include environmental governance, economic factors, values, and knowledge. Further research on trend-shifts, as well as bold action on underlying levers, is urgently needed to meet 2030 global conservation targets.

**Supplementary Information:**

The online version contains supplementary material available at 10.1007/s13280-024-02085-6.

## Introduction

To address climate change and global biodiversity loss, nations need clearly defined and scientifically defensible targets. Yet setting targets alone is insufficient, as these are often not met, as seen in the failure to achieve the 2020 Aichi Targets for biodiversity (Xu et al. 2021; Obura et al. 2023). Guidance on causes of failure and how to achieve the targets is therefore critical.

Many studies have investigated the feasibility of meeting climate targets (Tong et al. 2019). In contrast, the feasibility of meeting international conservation targets has received less attention (Obura et al. 2023). Such investigation requires identifying suitable indicators of progress, evaluating historical trends, and assessing the gap between the present state and trends and the targets, as well as identifying effective political, social, economic, or technological actions to reduce this gap.

Achieving global conservation targets requires transformative change (IPBES 2018). Transformative change is defined by the Intergovernmental Science-Policy Platform on Biodiversity and Ecosystem Services (IPBES) as a fundamental, system-wide reorganization across technological, economic, and social factors, including paradigms, goals, and values, needed for the conservation and sustainable use of biodiversity, good quality of life, and sustainable development. Understanding what has driven past successes and failures in conservation could significantly advance our ability to propel necessary transformative changes (Buxton et al. 2021; Grumbine and Xu 2021).

Here, we focus on three global conservation targets that are linked to land-use change, which is still the main driver behind biodiversity loss (IPBES 2018; Jaureguiberry et al. 2022). In particular, we selected (i) one target linked to the designation of protected areas, which have been considered so far as the cornerstone strategy of global biodiversity conservation (Maxwell et al. 2020), (ii) one target linked to deforestation and forest degradation, which is arguably the largest land-use change impacting global biodiversity (Giam 2017), and (iii) one target linked to ecosystem restoration, which is a direct response to the degradation and loss of ecosystems, and the importance of which has been recognized by United Nations by the declaration of 2021–2030 as the Decade on Ecosystem Restoration (Aronson et al. 2020).

The first target was set under the Kunming-Montreal Global Biodiversity Framework (KMGBF) of the UN Convention on Biological Diversity (CBD) and commits all parties to the CBD to protect at least 30% of terrestrial, inland water, and of coastal and marine areas by 2030 (CBD 2022). The second target directly links to the ‘Glasgow Leaders’ Declaration on Forests and Land Use, signed by 141 country representatives at the climate COP26, and commits signatories to “halt and reverse forest loss” by 2030 (UKCOP 2021). The third target is associated with the Bonn challenge for restoration, which sets a target of restoring 350 Mha of degraded and deforested landscapes by 2030 (Dave et al. 2017). These three complementary targets allow showcasing the different challenges faced in relation to monitoring progress and identifying the underlying levers that support it. Table 1 provides an overview of the targets.

**Table 1:** The three international conservation targets for 2030

Target	Organization and approval date	Objective
Target 3 in the Kunming-Montreal Global Biodiversity Framework	Convention on Biological Diversity (COP 15-2022)	To protect at least 30% of terrestrial, inland water, and of coastal and marine areas
Glasgow Leaders’ Declaration on Forests and Land Use	United Nations Framework Convention on Climate Change (COP 26-2021)	To halt and reverse forest loss
Bonn challenge for restoration	Launched by International Union for the Conservation of Nature (IUCN) and the government of Germany (2011)	To restore degraded and deforested landscapes by 350 Mha

Closing the gap between current trends and these targets requires actions that “bend the curve” (Mace et al. 2018) and bring about substantial shifts away from the business-as-usual (BAU) trends. Here, we define “trend-shifts” as those changes in the trajectory of a certain indicator that if sustained through time would allow the reaching of a specific target. A relatively gentle trend-shift may be sufficient if the bending of the curve starts soon. However, in cases of limited progress, a more abrupt trend-shift will be necessary as the deadline for the target is approached.

Research related to these three targets and the drivers that underpin progress towards them is highly uneven and not particularly oriented to identify past trend-shifts. Few studies assess the causal levers of protected area creation, with some studies focussing on related aspects such as wealth and education (McDonald and Boucher 2011), country size and power (Baldi 2020), historic and economic development (Brockington 2008), and motivations for conservation (Baldi et al. 2017). Similarly, research on the levers of restoration is limited, with a few studies identifying values, institutions, and financial instruments as levers (Aradóttir et al. 2013; Eger et al. 2020; Tedesco et al. 2023). Only avoided deforestation has been widely researched, including its proximate (*i.e.* direct) causes (via e.g. changing agricultural practices, economic instruments, and protected areas) and its indirect (i.e. underlying) causes (e.g. governance and institutional changes) (Busch and Ferretti-Gallon 2023), but still the amount of attention that different countries receive varies largely.

Global governance by goal setting holds potential for conservation but depends on several institutional factors as well as the broader context of the Great Acceleration of the Anthropocene (Steffen et al. 2015; Biermann et al. 2017; Folke et al. 2021). This context includes different dynamics and transitions such as population and economic growth (Jackson 2016; Simon 2019), urbanization (Bai et al. 2017), the agricultural transition (Alexander et al. 2015), and the forest transition (Pendrill et al. 2019), among others. These elements drive progress (or lack of) towards global conservation targets.

This article is structured as follows. First, a methodological section presents the datasets chosen and the analysis performed for the three targets. Second, a results section presents current progress, future projections, past trend-shifts, and the levers behind these. The next section discusses the main results and some key elements that could accelerate progress towards the targets in the short term, and is followed by a conclusions section.

## Methodology

Our methodological approach comprises three steps. First, in order to measure advancement towards the targets, we identified indicators and datasets of progress to date. For Target 3 of the KMGBF, which already defines different indicators that can be used to measure progress, this was relatively straightforward. When the target was not specific enough to assess progress with the use of a single indicator, or when no single indicator sufficed to evaluate progress (as in the case for deforestation and restoration), we selected several indicators and compared progress with these indicators. Second, to measure gaps between the targets and BAU trends, we extrapolated past trends based on relevant indicators forward to the year 2030. This approach is less complex than building scenarios such as the Shared Socio-economic Pathways (SSP) that integrate future changes of various elements to project different plausible futures (Leclère et al. 2020). However, it can be applied to any indicator linked to a particular target and can be more rapidly developed, which is necessary considering the short time-frame before the 2030 target deadline. To test the uncertainty of the projections we present, we performed several sensitivity analyses. Third, we identified past trend-shifts towards the targets by projecting past trends towards 2030 to see if the future projections of past trends reached the targets. Finally, we performed a structured literature review to identify the levers that have driven the identified trend-shifts towards the targets.

### Indicators and datasets

*Protected areas*: Following the KMGBF Monitoring Framework, we used the indicator on protected areas coverage from 1990 to 2023 from the World Conservation Monitoring Centre (WCMC), which include all IUCN Protected Area categories (I-VI) and Other Effective Conservation Mechanisms (OECMs) (UNEP-WCMC and IUCN 2024).

*Deforestation*: the Glasgow Declaration does not specify to which types of forests (e.g. primary vs nonprimary) or forest loss (gross vs. net zero) it refers (Nasi 2022; Gasser et al. 2022). Therefore, we used three datasets to assess the gap in progress towards the target: (1) global tree cover loss, (2) primary tropical forest loss, and (3) loss of Intact Forest Landscapes. The first two are available at the country level from 2001 to 2022 from Global Forest Watch (GFW), while data on Intact Forest Landscapes are available for the years 2000, 2013, 2016, and 2020 (GFW 2023). Datasets appear in Tables S1, S2 in the Supplementary Information.

**Table 2:** Pledges to the Bonn Challenge (Bonn Challenge Database, Accessed 21 Feb 2024), Gross forest gain (Potapov et al. 2022), and total planted forests (FAO 2023). Gross forest gain includes wildland, managed, and planted forests, agroforestry, orchards, and natural tree regrowth

	Pledges (Mha)	Gross forest gain 2000–2020 (Mha)	Total planted forests 2001–2020 (Mha)
Africa	128	12	2
Latin America	35	14	11
Asia and the Pacific	29	49	40
Europe	7	21	11
North America	34	33	13
TOTAL	234	131	77

*Restoration*: We could not identify any robust datasets that track global progress in restoration and through which past trend-shifts could be evaluated. As a compromise, we compiled three datasets that provide some insights into restoration progress towards the target. The first dataset consists of restoration pledges by governments. The other two datasets consist of data on gross forest gain (Potapov et al. 2022) and planted forests (FAO 2023). Planted forests include forest plantations, sometimes in the form of monocultures of exotic species and in some cases introduced into old-growth forests (Busch et al. 2015; Gaveau et al. 2019; 2022). Thus, it would be inaccurate to conflate reforestation with restoration (Parr et al. 2024). However, given the scarcity of data, this indicator helps understanding the limited progress towards the target, particularly when, as we show in the results section, the surface of planted forests is very far from what the target requests.

### Trend projections

To create projections forwards to 2030 at the global and national levels, we developed forecasts for each target using an Exponential Smoothing State Space model (ETS) (Hyndman et al. 2008) with triple exponential smoothing. ETS is not dominated by outliers and adjusts to changes in data over time, unlike other smoothing methods (some examples of other common and relevant methods are loess regression or curve fitting) that require a fixed model or assumptions about the structure of the data. ETS models have been applied to environmental and land use projections (Baykal et al. 2022; Siregar et al. 2017). We created one automated, one intermediate, and one extreme projection towards the targets by changing Alpha (a parameter used to measure the weight given to recent values in comparison with historic ones) and Beta (a parameter used to measure the weight given to the recent trend in comparison with the historical trend) which include different intervals of confidence, with 95% and 99.9% upper and lower confidence intervals. Supplementary Information shows the R code and libraries used to create them.

### Trend-shifts

We defined past trend-shifts as the changes in the trend of a variable in the direction of the target that, when projected towards the future, would result in hitting the target within the required time period. We identified past trend-shifts using the entire period of analysis (all existing data until the present time) as well as using shorter time-frames, to detect past trend-shifts that have not been sustained through time. This is presented for the establishment of marine protected areas (MPA) globally for which we used the 2006–2017 period, and for deforestation in Brazil, for which we used the 2001–2009 period. To limit our analysis of past-trend-shifts and subsequent literature review of levers that underpin them to a manageable number of countries, we focussed for deforestation on tropical forest loss only where we selected countries that account for 99% of total tropical primary forest loss for the 2001–2022 period (*n* = 39). This analysis should be expanded to other places outside the tropics, but we present it as an illustration of this approach. We could not identify trend-shifts for restoration as we could not identify robust datasets to track progress.

### Literature review

To identify levers that underpin past trend-shifts at the global and the country level, we undertook a review of peer-reviewed and grey literature for the specific trend-shifts identified. For that, we searched in the ISI Web of Science for terms that reflected the particular scale of analysis (global or country level, indicating the names of specific countries where we identified past trend-shifts), and terms linked to the particular target. For Target 3 of the KMGBF these included (“terrestrial”, “marine”, “protect*”, and “reserve*”) and for the Glasgow Leaders’ declaration these included (“deforest*” and “forest loss*”). To structure of review, we used the IPBES conceptual framework that includes as indirect drivers of change values and behaviours, demography and sociocultural aspects, economy and technology, institutions and governance, and conflicts and epidemics.

## Results

### Kunming-Montreal GBF Target 3 on protected area coverage: 30 × 30 target

**Fig. 1:**
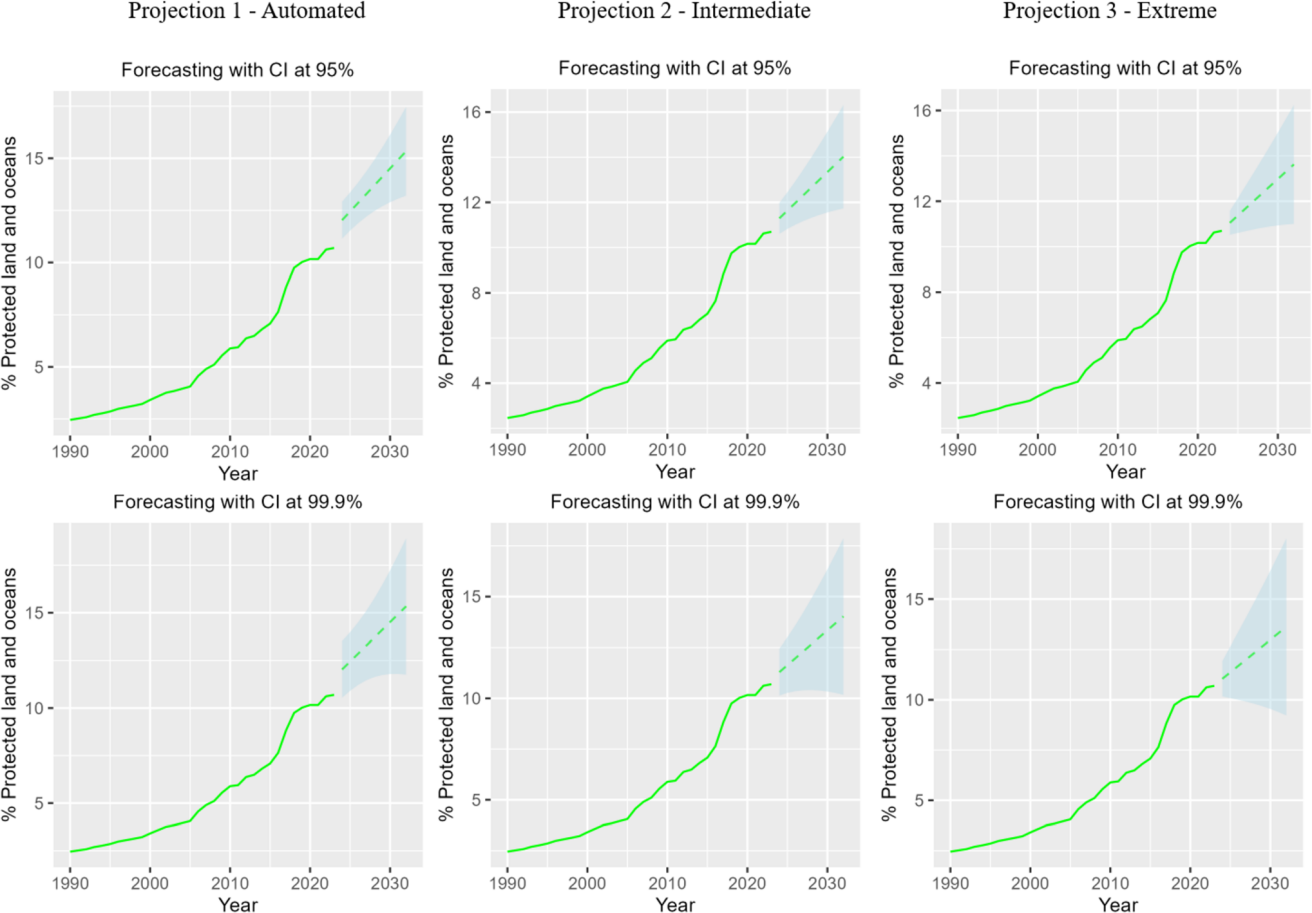
Projections of past trends for protected areas (coverage), under different confidence intervals (CI—95 and 99.9%) and Alpha and Beta values including an automated projection (Projection 1; Alpha (0.13); Beta (0.13)), an intermediate projection (Projection 2; Alpha (0.5); Beta (0.13)), and an extreme projection (Projection 3; Alpha (0.9); Beta (0.1759)). Alpha is a parameter used to measure the weight given to recent values in comparison with historic ones, and Beta is a parameter used to measure the weight given to the recent trend in comparison with the historical trend

**Fig. 2:**
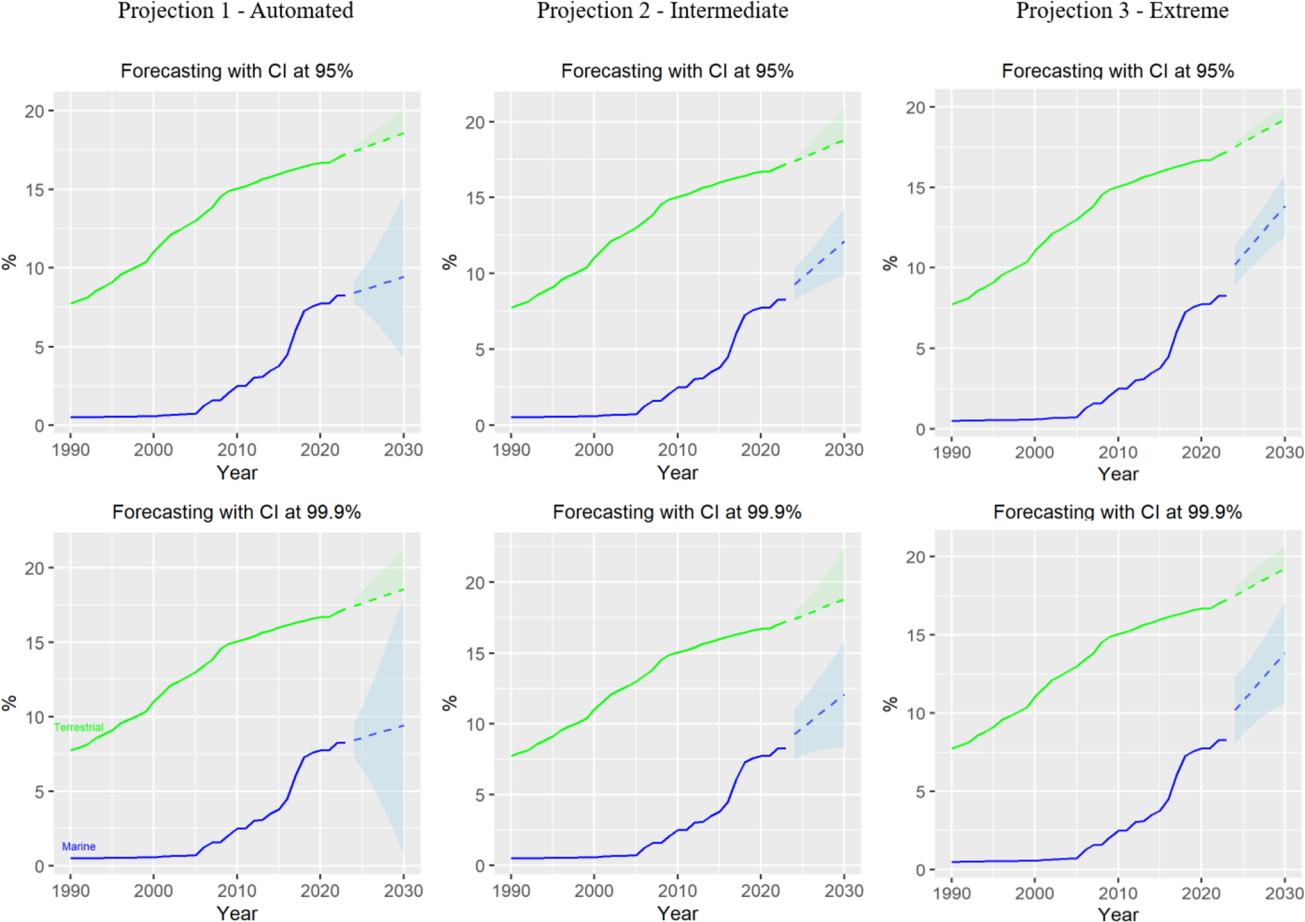
Projections of past trends for marine and terrestrial protected areas (coverage) under different confidence intervals (CI—95 and 99.9%) and Alpha and Beta values including an automated projection (Projection 1; Alpha (0.99); Beta (0.56 -Marine; 0.34-Terrestrial)), an intermediate projection (Projection 2; Alpha (0.4-Marine; 0.67-Terrestrial); Beta (0.1-Marine; 0.5 Terrestrial)), and an extreme projection (Projection 3; Alpha (0.1-Marine; 0.99-Terrestrial); Beta (0.1-Marine; 0.0001-Terrestrial))

For protected area coverage (including terrestrial and marine), none of the projections nor their upper confidence intervals (at 95 and 99.9%) towards 2030 indicate that the target will be reached (Fig. 1). The projection of past trends for terrestrial and marine protected area coverage separately also falls very short of meeting the 30% of protection designed by Target 3 of the GBF (Fig. 2). Figure 2 shows that a global past trend-shift occurred for marine protected areas (MPA) in the period 2006–2017, when MPA creation increased significantly (from 1 to 6% of coverage) around the world. The projection of the 1990–2017 trend towards 2030 indicates that the 2030 target for MPA is potentially within reach, as the upper confidence interval at 99.9% reaches a 30% of coverage. However, when we project the 1990–2023 trend, the projection falls short of the target because of the recent (2018–2023) slowdown in MPA declaration.

The sharp increase in MPA coverage that began in 2005 has been linked in previous research to a combination of key factors. From the IPBES classification of indirect drivers of change these mostly include values and behaviours, economic and technological aspects as well as institutions and governance. Specifically, these include legal factors concerning water sovereignty, a favourable policy environment, including international MPA targets (Lowry et al. 2009; Humphreys and Clark 2020), and evidence from influential scientific studies from the early 2000s, which linked MPA to greater catches in adjacent fisheries (Gell and Roberts 2003; Sale et al. 2005) and thus demonstrated that economic benefits exceeded costs of implementation and monitoring MPAs (Brander et al. 2020). Combinations of diverse factors like these have been proposed to explain previous transformative processes, but further research is still needed on how these elements interact with each other and over time through reinforcing mechanisms (Goddard et al. 2016; Palomo et al. 2021). Although causality cannot be assigned to any of these particular factors, it is important to consider them in combination to weigh up their individual and collective influence.

Transformative spill-over dynamics, such as replication across countries (Bennett et al. 2021), might also have driven the expansion of MPAs as countries have appeared to compete to establish the largest MPA (e.g. Papahānaumokuākea established by the USA and covering ca. 1.5 million km^2^). In the climate COP26, Ecuador, Colombia, Costa Rica, and Panama announced the enlargement of their MPA to achieve “the largest MPA of the north-western hemisphere, and possibly of the world”, as mentioned by Colombia’s ex-president through social media. It should be noted that, while such claims can help expand MPA coverage, they might also lead to policymakers neglecting the protection of biologically unique or vulnerable small places. Moreover, rushing the implementation of targets may reduce the conservation effectiveness (Agardy et al. 2016). Thus, a long-term planning approach that goes beyond the achievement of the 30 × 30 target is needed (Zabala et al. 2024).

### Glasgow Leaders declaration to halt and reverse forest loss

**Fig. 3:**
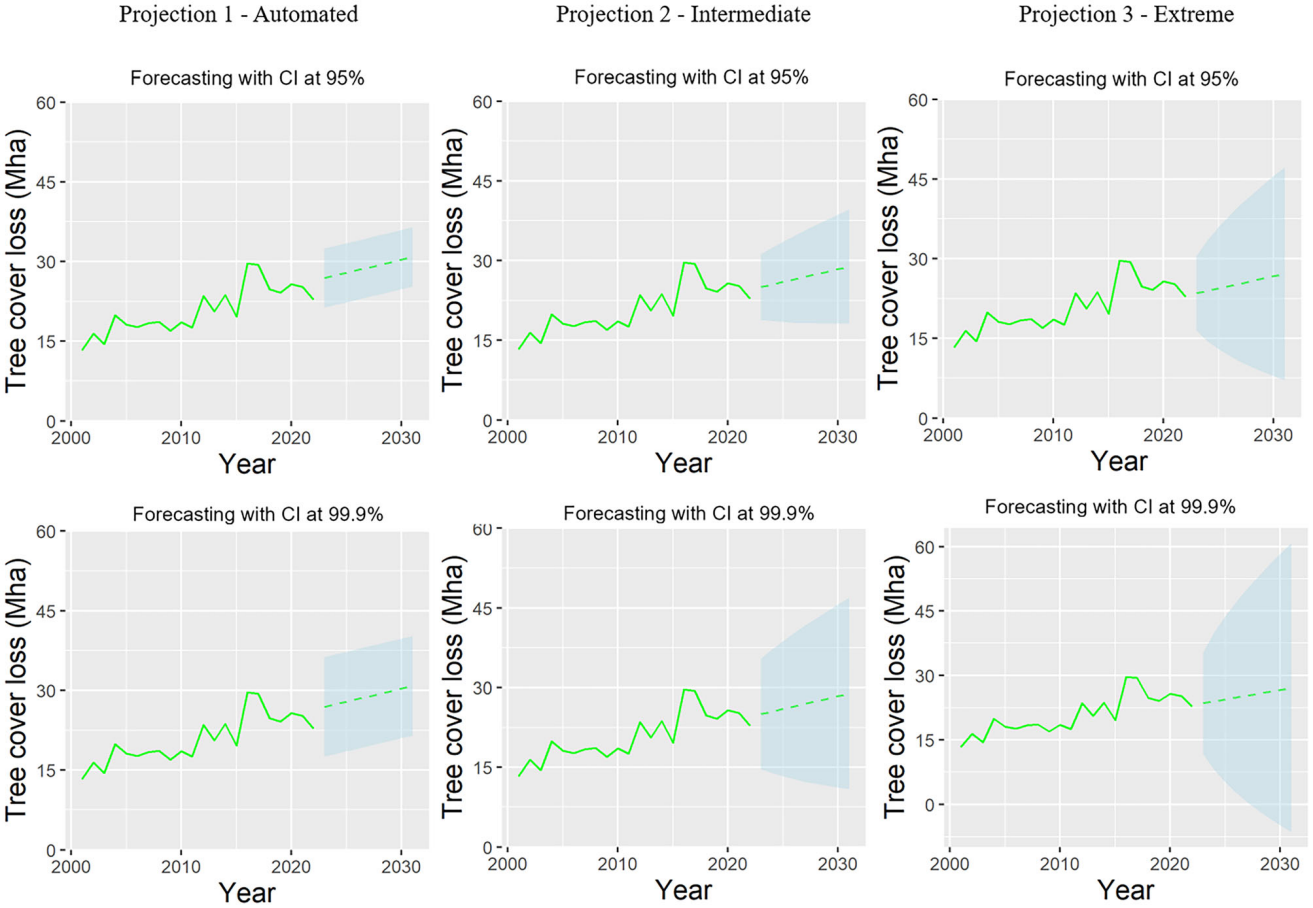
Projections of past trends for tree cover loss (Mha), under different confidence intervals (CI—95 and 99.9%) and Alpha and Beta values including an automated projection (Projection 1; Alpha (0.0001); Beta (0.0001)), an intermediate projection (Projection 2; Alpha (0.25); Beta (0.0001)), and an extreme projection (Projection 3; Alpha (0.9); Beta (0.01))

For deforestation, none of the projections of loss in global tree cover, intact forest landscapes, and tropical primary forest towards 2030 indicated that the target of halting deforestation would be reached (based on a confidence interval of 95%). When the confidence interval is increased to 99.9%, only the lower boundary of the confidence interval of the most optimistic of the three projections we developed suggests that the target could be met. However, the broad range of this confidence interval suggests that the likelihood of meeting the target is highly uncertain. Figure 3 presents these results for global tree cover loss, while tropical forest loss and intact forests landscape loss are presented in the Supplementary Information.

**Fig. 4:**
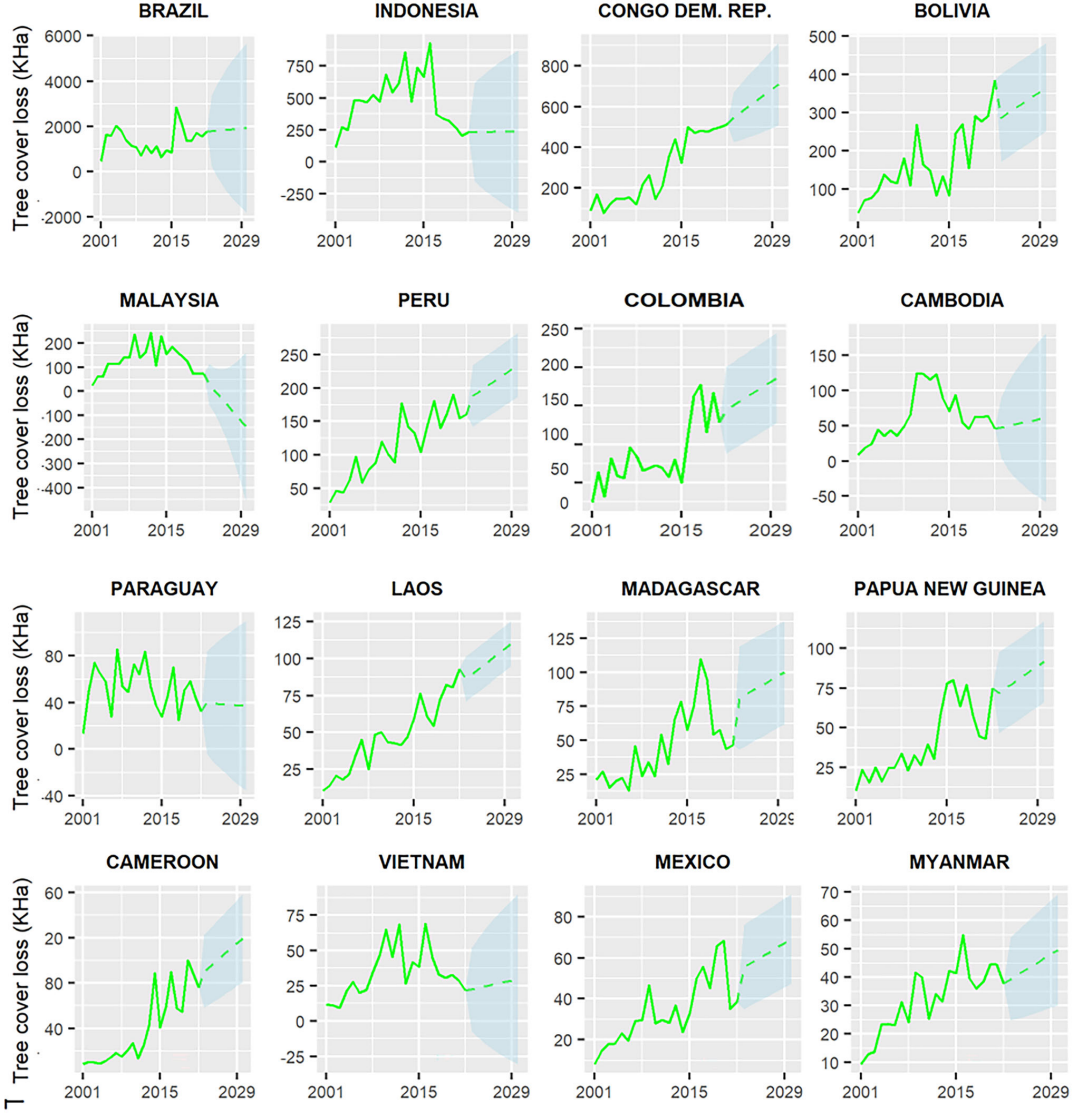
Projections of past trends for tropical primary forest loss (KHa). The figure shows past trends and projections towards 2030 of primary forest loss for the 16 countries that together account for 90% of total deforestation of tropical primary forests between 2001 and 2022. Countries are ordered according to the amount of (domestic) deforested forest area (Brazil: highest to Myanmar: lowest)

Given the high contextual diversity of avoided deforestation and its causes we investigated evidence for trend-shifts at the national scale. When the past trends of tropical primary forest loss of individual countries are projected to 2030, the data suggest that only Malaysia would meet the target of zero deforestation in 2030. For some other countries (Brazil, Indonesia, Cambodia, Paraguay, Vietnam, Guatemala, Argentina, Côte d'Ivoire, and Thailand), the lower boundary of the 95% confidence interval of the deforestation projection indicated that the zero-deforestation target would be met, although the confidence interval is too wide to allow strong conclusions. Figure 4 shows these results for countries representing 90% of cumulative tropical primary forest deforestation area (*n* = 16).

Most of the countries undergoing a trend-shift in reduced deforestation such as Malaysia, Indonesia, Cambodia, and Vietnam experienced a deforestation peak around 2011. Synchronies in peak-rates have been detected and discussed in terms of global resources use (Seppelt et al. 2014). In our case, 2011 is about the time when the prices of several agricultural commodities peaked in the international markets. The literature on factors that reduce deforestation also points to commodity prices. For instance, in Cambodia, Indonesia, and Malaysia, all major producers of rubber crops, the peak in the international price of rubber in 2011 and its subsequent price fall was a key factor contributing to decreasing deforestation rates (Grogan et al. 2019; Zhang et al. 2019). Similarly, in Indonesia, decreasing palm oil prices have been linked to reduced palm oil expansion and deforestation after 2012 (Gaveau et al. 2019), but other factors played out simultaneously, including the 2011 moratorium on clearing primary forests for logging or plantations (Government of Indonesia 2011; Busch et al. 2015) and the European demand-side restrictions on high-deforestation palm oil (Busch et al. 2022). Poverty reduction also played a role in Indonesia through conditional cash transfers (Ferraro and Simorangkir 2020) and income increases (Adilaa et al. 2021). Other factors than agricultural revenues contributed to reducing deforestation in Malaysia and Cambodia, including REDD + projects that enabled implementation of targeted community activities and rigorous monitoring and enforcement (Miyamoto 2020; Pauly et al. 2022). Observed deforestation reduction in Vietnam has been linked to conservation policies (Tran Quoc et al. 2023) and poverty reduction interventions (Van Khuc et al. 2018).

Exporting deforestation, which could help individual countries to reach their target but would undermine other countries in reaching targets, also played a major role. Many developed nations such as the USA and several European countries, as well as some fast-growing countries like China and India, have domestic net forest gains, partially due to exporting deforestation (Pendrill et al. 2019; Hoang and Kanemoto 2021). Malaysia, the only country whose projections suggest it could meet zero deforestation in 2030, has an 22 000 ha/yr of imported deforestation (as a comparison, Malaysia lost 71 926 ha of tropical primary forest in 2022, a much smaller figure than the 244 306 ha it lost in 2012) (Pendrill et al. 2019).

These examples from countries undergoing past trend-shifts show the diversity of indirect drivers behind them. While all main indirect drivers of change from the IPBES framework influenced deforestation, contextual diversity demands a case-by-case analysis of trend-shifts. For example, conflicts have been found to have both positive and negative effects on forest conservation in different regions (Butsic et al. 2015; Clerici et al. 2020).

The case of Brazil illustrates what a short-lived trend-shift can entail and which levers may underpin them. Between 2001 and 2009, Brazil was able to reduce its high deforestation levels, which peaked in 2004. The country achieved a large reduction in deforestation afterwards, particularly in the Amazon region. Projecting the trend of the 2001–2009 period to 2030 suggests that Brazil might have achieved zero deforestation if the trend of deforestation reduction had not been reversed, especially from 2013 onwards, and further aggravated during the Bolsonaro administration (2019–2022). The most often described indirect drivers behind this change have been related to institutions and governance, demographic, sociocultural, and economic factors. Land grabbing based on forest clearing has been historically and still is the main mechanism of deforestation in the Amazon, which usually takes place in undesignated public lands (Moutinho and Azevedo-Ramos 2023). Land grabbers illegally clear the forest and appropriate land usually by establishing low productivity cattle ranches. Two successive Brazilian presidential decrees in 2017 and 2019 declaring amnesties for illegal land grabbing made it possible to legally recognize land appropriated from 2005 to 2014 (Cardoso Carrero et al. 2022). In addition to these regulatory changes, during the Bolsonaro government there was a general dismantling of environmental policies, as well as a weakening of the state’s capacity to monitor, control, and enforce regulations with regards to forest loss (Abessa et al. 2021; Barbosa et al. 2021). The result was a great level of impunity for violations of environmental regulations. This backlash against environmental values in Brazil suggests that public policies and interventions aiming to reduce forest loss are vulnerable and can be reversed easily (Carvalho et al. 2019).

### The Bonn Challenge for restoration

As indicated above, we could not identify a robust dataset to track trend-shifts for restoration and its underlying levers. Table 2 serves as a rough estimate of progress towards the restoration target given the limitations of the three datasets identified. The fact that none of these three indicators of restoration coverage is close to the target set for 2030 supports the conclusion that there is a large gap in progress towards meeting the target, as previous studies have shown (Fagan et al. 2020; FAO and UNEP 2020).

Nonetheless, several restoration monitoring initiatives have recently started, such as the IUCN Restoration Barometer, which tracks restoration and works with governments to use the data it gathers, the World Resources Institute Global Restoration Initiative that monitors restoration globally and at multiple scales (from governmental jurisdictions to individual projects), and Restor, a data sharing platform that tracks restoration and conservation interventions (Crowther et al. 2022).

Restoration pledges by governments have increased considerably to over 200 Mha worldwide. However, it is not clear to what extent or how this figure could be reached in practice (Fagan et al. 2020). As of 2019, only 18% of the goal to restore 150 Mha by 2020 had been achieved (NYDF 2019). In 2022, there were only 14 Mha under restoration as reported by 18 countries, a figure far from the target, although this figure may increase as more countries report to the IUCN Barometer (IUCN 2022) (e.g. China is not a reporting country in the IUCN 2022 barometer, but has implemented large-scale restoration projects (Chen et al. 2019)). In addition, clarity is needed on what counts as restoration. For instance, commitments to the Bonn Challenge by countries often include forest plantations, the regeneration of natural forests, and agroforestry (Dave et al. 2017). Silviculture and natural regeneration are the largest forms of forest landscape restoration activities by area (FAO and UNEP 2020), but the restoration of other ecosystem types should be included in monitoring efforts as well (e.g. grasslands—Bardgett et al. 2021; Buisson et al. 2022).

## Discussion

Our projections of past trends in protected areas and forest loss, along with the evidence for restoration, indicate that without transformative change, the 2030 conservation targets will not be achieved. Our findings highlight the critical importance of identifying past trend-shifts in understanding and driving transformative change, especially within the context of global conservation goals. The recognition of rapid shifts towards conservation—such as the expansion of MPAs and reductions in deforestation in specific countries—provides valuable insights into the mechanisms that can promote transformative change. These past successes illustrate how integrated approaches can facilitate the achievement of ambitious environmental objectives (Leclère et al. 2020; Leadley et al. 2022; Echeverri et al. 2023). The significant trend-shifts in MPA creation, particularly from 2006 to 2017, were largely driven by a combination of international agreements, legal frameworks, and a growing body of evidence demonstrating the economic benefits of MPAs. These factors acted as key levers of change, underscoring the role of governance and knowledge in enabling transformative shifts, as highlighted by Furumo and Lambin (2021). A key policy implication of these findings is that integrated actions should be prioritized in both planning and management (Díaz et al. 2020).

Among this diversity of actions, there are several core elements that could accelerate the pace of progress towards the targets in the near future. The recently established Other Effective area-based Conservation Mechanisms (OECMs) could prove fundamental to reaching the 30 × 30 target since they can be established over sustainably managed areas (Dinerstein et al., 2019; Maxwell et al. 2020; Gurney et al. 2021). Through OECMs, Indigenous Peoples and their territories, often marginalized by national governments despite the key contribution they have provided to conservation, could meaningfully contribute towards the 30 × 30 target in the coming years (Palomo et al. 2014; Garnett et al. 2018; Tauli-Corpuz et al. 2020; Sze et al. 2022). The recently approved UN treaty to protect the high seas could increase MPA coverage greatly, particularly considering that only a few, very large and remote MPA were the main contributors to the large increases in area of MPA coverage in previous years (Devillers et al. 2015; Jarvis and Young 2023). While these efforts to reach the 30 × 30 target are useful, effective and equitable measures to avoid leaving existing large protected areas under-resourced and under increased pressure need to be put in place (Gill et al. 2017; Coad et al. 2019; Visconti et al. 2019; Claudet et al. 2020).

The large gaps identified also suggest that a different and more effective environmental governance architecture is needed to achieve progress towards the targets. This is supported by the fact that past international treaties have often failed to produce their intended effects (Biermann et al. 2022; Hoffman et al. 2022). As suggested by Mace et al. (2018) and others (e.g. Xu et al. 2021), a recurring global process to evaluate progress and commitments by nations, as is in place for climate change targets through the Paris Agreement, is needed for conservation targets. Adequate funding to support progress is a key element, but the US$ 200 billion per year assigned to the Global Biodiversity Framework Fund is still considered far from what is needed to achieve GBF targets (Deutz et al. 2020; Xu et al. 2021). Historical responsibility for environmental degradation and the inequitable distribution of conservation costs lead to the argument that countries in the Global North should provide increased support to the Global South (Weinzettel et al. 2018; Fanning et al. 2022; Waldron et al. 2022).

## Concluding remarks

The high level of ambition of 2030 global conservation targets for protected areas, deforestation, and restoration is a remarkable success of the global environmental community. However, past failures in the achievement of global conservation targets demand a detailed and timely assessment of progress towards these targets.

Here, we provide an assessment of progress for three global conservation targets and project past trends towards 2030 to explore the feasibility of their achievement. Our projections show that in a business as usual scenario, none of these targets would be met.

Past trend-shifts, or rapid progress towards conservation targets, could help understand what drives transformative change towards them. Our analysis of past historical trends identifies a few rapid trend-shifts for marine protected area creation and for avoided deforestation in certain countries. These trend-shifts have been underpinned by a diversity of indirect drivers of change, including environmental governance, economic factors, values, and knowledge.

Measuring progress towards international targets and providing guidance to achieve them is a core aspect of global environmental research that has multiple challenges. The complexity, nonlinearity, and regime shifts that characterize social-ecological systems make projecting future trends towards targets challenging. Nonetheless, the very wide gap that these projections show in terms of reaching the targets confirms the need to urgently speed up progress towards them.

## Supplementary Information

Below is the link to the electronic supplementary material.

Supplementary file1 (PDF 578 kb)
