# Publisher Correction: Business-as-usual trends will largely miss 2030 global conservation targets

**DOI:** 10.1007/s13280-024-02114-4

**Published:** 2024-12-04

**Authors:** Ignacio Palomo, Alberto González-García, Paul J. Ferraro, Roldan Muradian, Unai Pascual, Manuel Arboledas, James M. Bullock, Enora Bruley, Erik Gómez-Baggethun, Sandra Lavorel

**Affiliations:** 1 Univ. Grenoble-Alpes, IRD, CNRS, Grenoble INP, INRAE, IGE, 38000 Grenoble, France; 2 Carey Business School and Department of Environmental Health and Engineering, Johns Hopkins University, Baltimore, MD USA; 3 Faculty of Economics, Universidade Federal Fluminense, Niterói, Rio de Janeiro Brazil; 4 Centre for Environment and Development, University of Bern, 3012 Bern, Switzerland; 5 Basque Centre for Climate Change, BC3, 48940 Leioa, Bizkaia Spain; 6 Ikerbasque, Basque Foundation for Science, 48009 Bilbao, Bizkaia Spain; 7 Universidad de Jaén, Jaén, Spain; 8 UK Centre for Ecology & Hydrology (UKCEH), Wallingford, UK; 9 Department of International Environment and Development Studies (Noragric), Faculty of Landscape and Society, Norwegian University of Life Sciences (NMBU), PO Box 5003, 1432 Ås, Norway; 10 Norwegian Institute for Nature Research (NINA), Gaustadalléen 21, 0349 Oslo, Norway; 11 Laboratoire d’Ecologie Alpine, UMR 5553, CNRS–UGA-USMB, CS 40700, 38058 Grenoble Cedex 9, France

**Publisher Correction: Ambio** 10.1007/s13280-024-02085-6

In the original published article, one of the author names “Roldan Muradian” was mistakenly written as “Rodan Muradian” during the production process.

The original article has been corrected.

