# Supplementary material for: Business-as-usual trends will largely miss 2030 global conservation targets
Source: Ambio. 2024 Nov 7;54(2):212–24. doi: 10.1007/s13280-024-02085-6 (PMC11662131; doi:10.1007/s13280-024-02085-6)
Supplement: Supplementary file 1 — Supplementary file1 (PDF 578 kb) [file 13280_2024_2085_MOESM1_ESM.pdf]

Ambio

Supplementary Information
This supplementary information has not been peer reviewed

Title: **Business-as-usual trends will largely miss 2030 global conservation targets**

This perspective presents a preliminary assessment of progress towards three international targets as well as some guidance based on previous trend-shifts and a literature review. As such, it does not intend to be exhaustive, as for example it does not include other important conservation targets such as Land Degradation Neutrality (Cowie et al., 2018). Rather, we intend to (1) raise awareness of the slow pace of global progress towards these three targets considering the limited available time to achieve them; and (2) highlight the need for improved monitoring of progress towards targets as well as for research on the levers that have underpinned past trend-shifts.

Measuring progress towards international targets and providing guidance to achieve them is a core aspect of global environmental research that has multiple challenges. The complexity, non-linearity and regime shifts that characterise social-ecological systems (Adams and Sandbrook, 2013; Folke et al., 2021), and the sometimes hard-to-predict changes in land use systems (Meyfroidt et al., 2022), make projecting future trends towards targets challenging. Moreover, unexpected changes and rapid transitions have happened in the past (Rudel et al., 2020). Therefore, caution is needed concerning the future projections we have presented. Nonetheless, the very wide gap that projections show in terms of reaching the targets confirm the need to rapidly speed up progress.

We acknowledge that this is an exploratory approach that does not incorporate potential future changes in those elements that could influence future trends of these indicators. For instance, these could include direct drivers of change such as climate change, but also indirect drivers of change such as dynamics of values about nature, awareness of future risks, changes in lifestyles or political changes, just to name a few. The inclusion of shallow and deep leverage points into studies about futures also faces many challenges. Previous models designed to represent social elements in land-based sectors have mostly included aspects of human behaviour linked to shallow leverage points, such as economic-based decision-making influencing land use changes (Brown et al., 2017). Economic theories of "rational actors" underpin many of such models, ignoring other disciplines that seek to understand the complexity of decision making (Groeneveld et al., 2017). Verburg et al. (2019) arrived at a similar conclusion and suggested the need for a new generation of land use models that incorporate deeper leverage points and perspectives from the social sciences (Malek and Verburg, 2020).

The data we have used for exploring progress towards the targets has limitations. For protected areas, we relied on the global long-term series of protected areas creation of the World Database of Protected Areas. This datasets contains certain limitations regarding the year of the establishment of certain protected areas as well as the Protected Area Downgrading, Downsizement and Degazettement (PADDD) (Mascia and Pailler, 2011). Historical country-level data is provided for the years 2016-2023 by the World Bank, but we considered this short time frame inadequate to evaluate past trend shifts in the long history of protected area creation. For deforestation, the future projections we have presented are based on the loss of global tree cover, tropical primary forest and intact forest landscapes. In these data sets, "tree cover" is defined as "all vegetation greater than 5 meters in height, and may take the form of natural forests or plantations across a range of canopy densities". "Primary forests" are defined as "mature natural humid tropical forest cover that has not been completely cleared and regrown in recent history". Intact Forest Landscapes, are identified by GFW as "the last remaining unfragmented forest landscapes, large enough to retain all native biodiversity and showing no signs of human alteration as of the year 2016". "Loss" indicates the removal or mortality of tree cover due to a variety of factors, including mechanical

harvesting, fire, disease, or storm damage. As such, "loss" does not strictly equate to deforestation. To limit our analysis of past-trend shifts and subsequent literature review of levers that underpin them to a manageable number of countries, we focused for deforestation on tropical forest loss only. Moreover, outside the tropics, forest loss is mainly driven by forestry and wildfires and it is therefore harder to link past trend-shifts to scientific evidence from the literature of indirect drivers of change that reduce deforestation such as changes in commodity prices or specific environmental governance such as incentives or legislation (Curtis et al., 2018). Tropical forest loss is largely linked to commodity-driven deforestation and shifting agriculture and allows a more specific evaluation of strategies that can help reduce deforestation (Curtis et al., 2018). Data limitations have not allowed us to perform similar analysis in other specific forest types affected by deforestation such as subtropical dry woodlands or temperate primary forest (Buchadas et al., 2023). A deeper analysis could benefit from using deforestation rates per year instead of absolute deforestation due to shifting forest cover baselines since the percentage of remaining forest would diminish through time in the case of constant deforestation. Here we have focused on three particular targets that relate to the conservation of biodiversity, but we did not differentiate between different components of biodiversity (ie genetic diversity, functional diversity) and how these are affected by protected areas, deforestation or ecosystem restoration. We also did not analyse the conservation of biodiversity directly, which is still fundamental considering that protected areas do not guarantee the full conservation of biodiversity within their boundaries and that the level of biodiversity conservation in restoration projects differs substantially (Lawrence et al., 2012; Seddon et al., 2019).

Drawing lessons from historical trends has also limitations because particular contexts from the past might not be replicable in the future and recreating conditions to scale up success stories could prove challenging. For example, it would be challenging to draw lessons from the trend-shift in MPA that could be applicable to terrestrial protected areas due to their very different contexts. Nonetheless, lessons from MPA could help advance future MPA establishment, and the insights we provide on the reduction in forest loss for some countries might be applicable to other countries in similar contexts. In addition, interdisciplinary knowledge synthesis of what has worked in the past is fundamental to create the enabling conditions for the emergence of future factors as well as to produce generalizable knowledge (Magliocca et al., 2018). Moreover, highlighting previous environmental successes could help society unite to act to drive positive change (McAfee et al., 2019). Future research should explore further the diverse values of nature, that are not well covered in the literature on land use change and protected areas, deforestation and restoration, but that certainly play a major role in environmental governance (IPBES, 2022; Pascual et al., 2023). Similarly, aspects of power, and how changes in power could reduce forest loss, should receive further attention in the literature (Bennett et al., 2018).

We have not generally applied a standard method to all the datasets to identify past trend shifts, nor defined a minimum time period for these. For future studies that further develop approaches to evaluate trend shifts, it would be useful to evaluate them together with their associated underpinning levers. Moreover, we have not explored here the links between the three conservation targets themselves beyond the already mentioned teleconnections and exported deforestation, as this would require a different approach. Nonetheless, as we have highlighted it is critical to consider them, and other works have assessed these teleconnections globally (Hoang and Kanemoto, 2021; Pendrill et al., 2019) and at coarse scales for example in terms of deforestation leakage of forested protected areas (Ford et al., 2020). Finally, we

have not assessed to what extent trend shifts have occurred as a response of the targets being set, which is a fundamental question for global environmental governance.

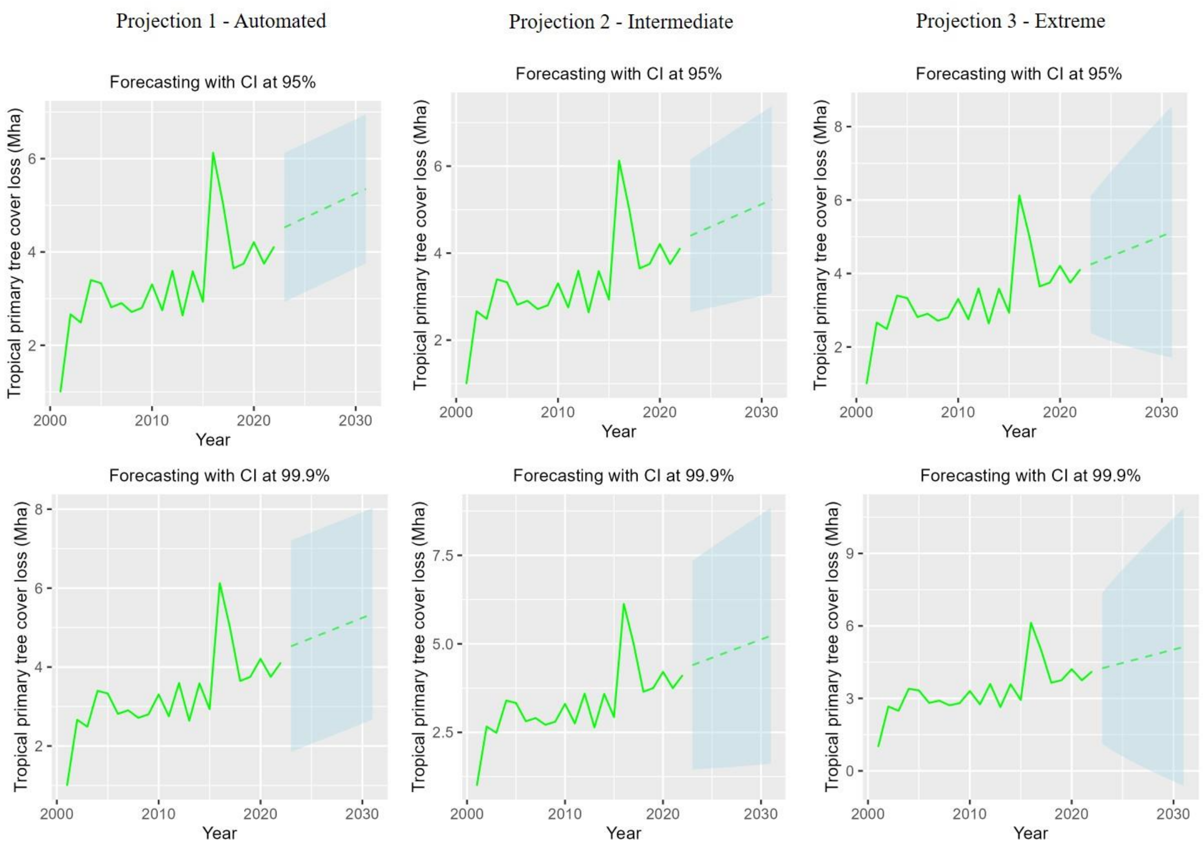

Figure S1. Projections of past trends for tropical forest loss (Mha), under different confidence intervals (CI – 95 and 99.9%) and Alpha and Beta values including an automated projection (Projection 1; Alpha (0.0001); Beta (0.0001)), and intermediate projection (Projection 2; Alpha (0.25); Beta (0.0001)) and an extreme projection (Projection 3; Alpha (0.9); Beta (0.01)).

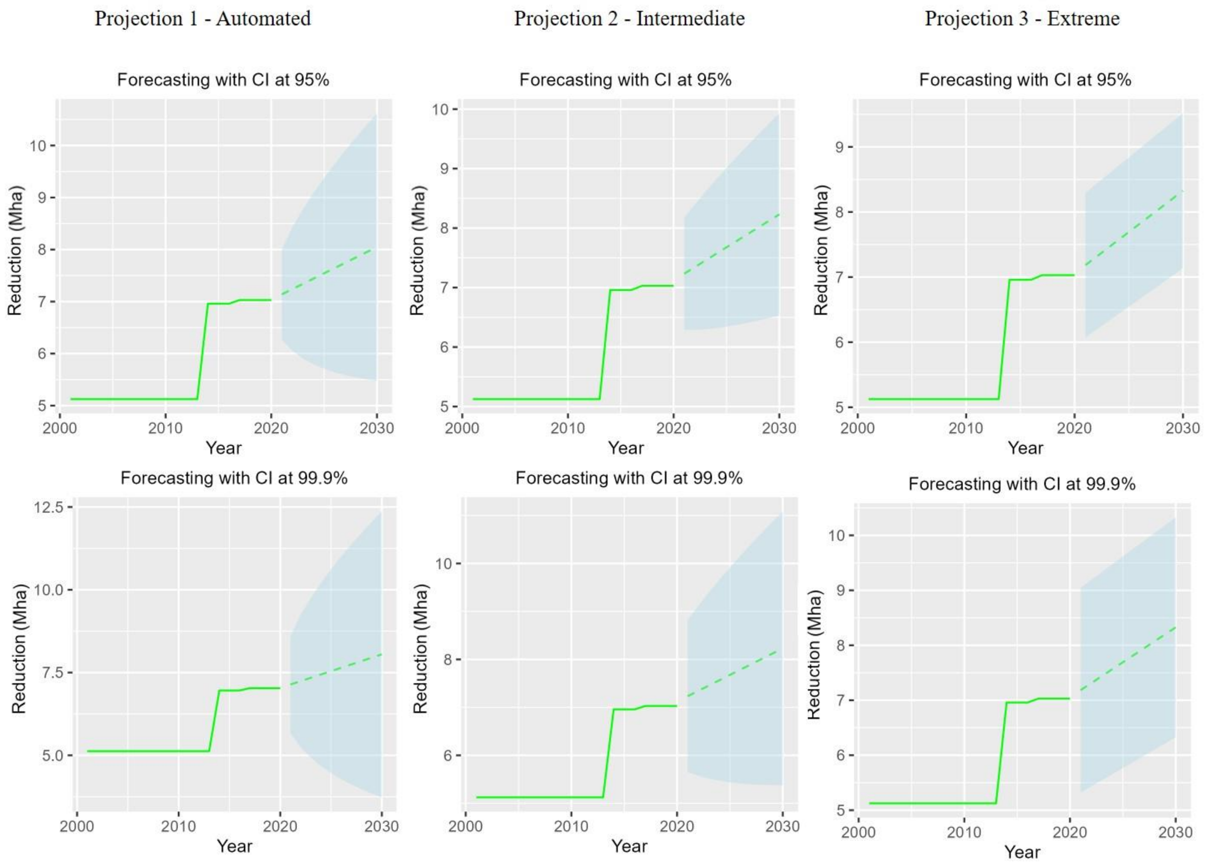

Figure S2. Projections of past trends for intact forest landscapes loss (Mha), under different confidence intervals (CI – 95 and 99.9%) and Alpha and Beta values including an automated projection (Projection 1; Alpha (0.0001); Beta (0.0001)), and intermediate projection (Projection 2; Alpha (0.25); Beta (0.0001)) and an extreme projection (Projection 3; Alpha (0.9); Beta (0.01)).

References

Adams, W. M. C. Sandbrook. 2013. Conservation, evidence and policy. Oryx 47(3): 329-335.

Bennett, A., A. Ravikumar, and H. Paltán. 2018. The Political Ecology of Oil Palm Company-Community partnerships in the Peruvian Amazon: Deforestation consequences of the privatization of rural development. *World Development* 109: 29-41.

Brown, C., P. Alexander, S. Holzhauer, and M.D. Rounsevell. 2017. Behavioral models of climate change adaptation and mitigation in land-based sectors. *Wiley Interdisciplinary Reviews: Climate Change* 8(2): e448.

Buchadas, A., M. Jung, M. Bustamante, A. Fernández-Llamazares, S.T. Garnett, A.S. Nanni, N. Ribeiro, P. Meyfroidt, et al. 2023. Tropical dry woodland loss occurs disproportionately in areas of highest conservation value. *Global Change Biology* 29(17): 4880-4897.

Cowie, A. L., B.J. Orr, V.M.C. Sanchez, P. Chasek, N.D. Crossman, A. Erlewein, G. Louwagie, M. Maron, et al. 2018. Land in balance: The scientific conceptual framework for Land Degradation Neutrality. *Environmental Science & Policy* 79: 25-35.

Curtis, P.G., C.M. Slay, N.L. Harris, A. Tyukavina, and M.C. Hansen. 2018. Classifying drivers of global forest loss. *Science* 361(6407): 1108-1111.

Folke, C., S., Polasky, J. Rockström, V., Galaz, F. Westley, M. Lamont, M. Scheffer, H. Österblom, et al. 2021. Our future in the Anthropocene biosphere. *Ambio* 50: 834-869. https://doi.org/10.1007/s13280-021-01544-8.

Ford, S.A., M.R. Jepsen, N. Kingston, E. Lewis, T.M. Brooks, B. MacSharry, and O. Mertz. 2020. Deforestation leakage undermines conservation value of tropical and subtropical forest protected areas. *Global Ecology and Biogeography* 29(11): 2014-2024.

Groeneveld, J., B. Müller, C.M. Buchmann, G. Dressler, C. Guo, C., N. Hase, F. Hoffman, F. John, et al. 2017. Theoretical foundations of human decision-making in agent-based land use models–A review. *Environmental modelling & software* 87: 39-48.

Hoang, N.T., and K. Kanemoto. 2021. Mapping the deforestation footprint of nations reveals growing threat to tropical forests. *Nature Ecology and Evolution* 5(6): 845-853.

IPBES. 2022. Summary for policymakers of the methodological assessment of the diverse values and valuation of nature of the Intergovernmental Science-Policy Platform on Biodiversity and Ecosystem Services. Pascual et al., eds. IPBES secretariat, Bonn, Germany.

Laurance, W. F., D. Carolina Useche, J. Rendeiro, M. Kalka, C.J. Bradshaw, S.P. Sloan, S.G. Laurance, M. Campbell, et al. 2012. Averting biodiversity collapse in tropical forest protected areas. *Nature* 489(7415): 290-294.

Magliocca, N.R., E.C. Ellis, G.R. Allington, A. De Bremond, J. Dell'Angelo, O. Mertz, P. Messerly, P. Meyfroidt, et al. 2018. Closing global knowledge gaps: producing generalized knowledge from case studies of social-ecological systems. *Global Environmental Change* 50: 1-14.

Malek, Ž., P.H. Verburg. 2020. Mapping global patterns of land use decision-making. *Global Environmental Change* 65: 102170.

Mascia, M. B., and S. Pailler. 2011. Protected area downgrading, downsizing, and degazettement (PADDD) and its conservation implications. *Conservation letters 4*(1): 9-20.

McAfee, D., Z.A. Doubleday, N. Geiger, and S.D. Connell. 2019. Everyone loves a success story: optimism inspires conservation engagement. *Bioscience* 69(4): 274-281.

Meyfroidt, P., A. De Bremond, C.M. Ryan, E. Archer, R. Aspinall, A. Chhabra, G. Camara, E. Corbera, et al. 2022. Ten facts about land systems for sustainability. *Proceedings of the National Academy of Sciences* 119(7): e2109217118.

Pascual, U., P. Balvanera, C.B. Anderson, R. Chaplin-Kramer, M. Christie, D. González-Jiménez, A. Martin, C.M. Raymond, et al. 2023. Diverse values of nature for sustainability. *Nature 620*(7975): 813-823.

Pendrill, F., U.M. Persson, J. Godar, T. Kastner. 2019. Deforestation displaced: trade in forest-risk commodities and the prospects for a global forest transition. *Environmental Research Letters* 14(5): 055003.

Rudel, T.K., P. Meyfroidt, R. Chazdon, F. Bongers, S. Sloan, H.R. Grau, T. Van Holt, and L. Schneider. 2020. Whither the forest transition? Climate change, policy responses, and redistributed forests in the twenty-first century. *Ambio* 49: 74-84. https://doi.org/10.1007/s13280-018-01143-0.

Seddon, N., B. Turner, P. Berry, A. Chausson, and C.A. Girardin. 2019. Grounding nature-based climate solutions in sound biodiversity science. *Nature Climate Change 9*(2): 84-87.

Verburg, P.H., P. Alexander, T. Evans, N.R. Magliocca, Z. Malek, M.D. Rounsevell, and J. van Vliet. 2019. Beyond land cover change: towards a new generation of land use models. *Current Opinion in Environmental Sustainability* 38: 77-85.

Datasets

Protected Areas

Table S1. Accumulated surface for terrestrial and marine protected areas and Other Effective Conservation Mechanisms and associated coverage.

Year	Terrestrial surface (Km^2)	Marine surface (Km^2)	Land Coverage (%)	Marine Coverage (%)
1990	10421720	1788639	7,75	0,49
1991	10690997	1836570	7,95	0,51
1992	10953300	1876973	8,14	0,52
1993	11515673	1891841	8,56	0,52
1994	11857678	1913379	8,81	0,53
1995	12251551	1956095	9,11	0,54
1996	12859538	1977213	9,56	0,55
1997	13222097	2001941	9,83	0,55
1998	13563975	2034089	10,08	0,56
1999	13944045	2067754	10,36	0,57
2000	14859268	2104125	11,04	0,58
2001	15535290	2296995	11,55	0,63
2002	16280423	2406245	12,10	0,66

2003	16657553	2439311	12,38	0,67
2004	17078161	2557635	12,69	0,71
2005	17507281	2658893	13,01	0,73
2006	18097124	4571858	13,45	1,26
2007	18603564	5748867	13,83	1,59
2008	19555267	5829580	14,53	1,61
2009	20030748	7559697	14,89	2,09
2010	20246021	9014531	15,05	2,49
2011	20459278	9076928	15,21	2,50
2012	20716469	10951201	15,40	3,02
2013	21048755	11194508	15,64	3,09
2014	21237634	12611495	15,78	3,48
2015	21500105	13678949	15,98	3,77
2016	21722736	16240150	16,14	4,48
2017	21927184	21960605	16,30	6,06
2018	22110506	26330389	16,43	7,26
2019	22332517	27480898	16,60	7,58
2020	22454521	28054196	16,69	7,74
2021	22454710	28054196	16,69	7,74
2022	22847496	29938974	16,98	8,26
2023	23183885	29975220	17,23	8,27

Deforestation

Table S2. Surface of tropical primary forest loss (Ha) for the 39 countries that account for 99% of total tropical primary forest loss in the 2001-2022 period.

Country	2001	2002	2003	2004	2005	*2006*	2007	2008	2009	2010	2011	2012	2013	2014	2015	2016	2017	2018	2019	2020	2021	2022
BRA	465543	1621765	1570576	2016477	1824425	*1415580*	1149563	1075146	700169	1153025	803049	1116088	632094	940905	828870	2830977	2134649	1347133	1361094	1704092	1548658	1772690
IDN	114394	271745	248436	479153	478406	*468382*	525993	469407	683828	540976	613906	855534	472991	736798	666642	928660	373255	339888	323646	270057	202905	230003
COD	92020	168993	78138	123521	149894	*146457*	154991	121474	218277	265180	148639	211234	351217	439345	322814	499560	471868	481248	475246	490613	499059	512672
BOL	36530	70601	77167	96611	137831	*118804*	114376	180575	108163	267751	162625	148294	82290	133268	83299	246088	270346	154489	290499	276883	291380	385568
MYS	24068	61820	60416	113422	114496	*113080*	142068	139246	235835	139647	161191	244307	106041	230429	154354	185215	162417	144571	120180	72977	72234	71927
PER	28699	46059	43733	62035	97399	*58813*	77992	88797	120186	100970	88886	177236	142870	133107	104864	142720	181090	140185	161590	190199	154279	160991
COL	24082	63302	32050	81695	58906	56051	95539	83619	65824	68739	72601	69587	57713	80036	49643	108566	161945	176977	115090	166485	128563	128455
KHM	8908	18576	24054	44063	35969	43256	35533	48734	65268	124772	124149	115205	123195	89450	70632	93616	53750	45538	62886	62295	63982	45376
PRY	13534	50509	74444	65085	57705	27949	85752	53657	49612	72835	64119	83806	53487	38157	28271	45863	70056	25298	50818	58572	44749	32588
LAO	10263	13700	20471	17947	21601	33978	44922	24927	48415	49980	43373	42486	41318	46969	58992	76479	60711	54581	72038	82239	80939	93089
MDG	21194	27045	15333	20283	22517	12821	45970	23616	34019	23825	54167	32423	65211	78699	57831	74991	109612	94785	54257	57748	43745	46546
PNG	10238	23530	15366	25103	16365	24785	25053	33901	23180	32760	26450	39701	30630	58478	77941	80232	63581	77266	57963	44865	43338	74970
CMR	8287	10612	9967	9049	11079	14846	18347	15038	19722	26815	13506	25417	42764	89176	40994	58602	89783	57935	54422	100295	88756	76312
VNM	12002	11030	9522	21113	27779	20138	22282	34398	46798	64629	45403	68337	26715	41830	38582	68855	45012	33386	30894	32620	29007	21668
MEX	8045	14329	17925	18119	23058	19398	28987	29568	46684	27933	29571	28225	36628	23837	32682	50145	55702	45321	65826	68423	35129	38391
MMR	9308	12939	13768	23286	23421	23066	31292	24124	41647	40067	25248	34051	31384	42157	41429	54825	39689	35913	38310	44445	44566	37798
NIC	1853	5755	5124	3183	11310	8820	36160	8021	16023	30518	14501	7265	14016	23321	12543	47410	150585	32009	33913	52626	54031	22636
VEN	10438	11323	20775	15924	15565	14244	26116	19859	23435	25809	15590	22125	15349	20609	15546	84705	43759	30169	58827	53702	22709	19811
GTM	3979	16082	24305	17270	33388	18337	46827	29728	37529	22750	17213	27789	19870	18237	16722	63225	22036	18330	30255	39451	15160	12218
HND	4747	7344	8856	7115	14158	14166	12660	14680	22180	21407	20323	10008	16970	22265	15366	46024	37700	32645	32911	41688	36601	30126
ARG	5980	9317	14448	28084	31424	24101	18688	47052	20819	31311	17366	24810	56675	19290	10542	15242	17189	9494	8965	20834	11905	21382
COG	7399	9974	9875	4996	8608	11788	13689	5893	7705	20598	13755	14806	24818	31143	18197	43659	26703	26847	30648	26296	19571	26089
IND	5542	11718	10846	19166	15148	15125	17208	20702	17112	11361	16287	18804	14399	21942	20997	30936	29563	19310	17347	20809	22038	21839
CIV	14045	24186	16573	11229	6007	16768	17323	12553	14683	17374	29740	23086	18853	36119	15592	16912	20418	25669	11649	12959	6615	8588
LBR	2081	7330	4821	1782	2168	6005	7640	5891	13356	4633	5797	11268	16258	18963	25850	19754	24957	27854	23408	31822	24821	30365

GAB	5118	7002	10674	5699	10816	10495	10584	9314	11766	6237	8689	9508	25802	29149	15816	18886	22916	12251	13816	10629	8858	14511
ECU	4701	5693	3379	5436	6205	6438	6995	8953	8112	8491	11175	16354	11590	6330	8472	13198	21085	13220	12231	19747	12472	21014
CAF	3940	7005	3026	4096	5466	5684	8400	5293	8869	11148	9501	10780	6481	8935	5064	20130	15443	7742	11530	14742	19709	13242
PHL	1655	3499	4275	7078	6333	7144	8753	5656	6946	8880	4919	6889	10835	11484	6477	13387	13698	9222	7586	7751	6829	27288
AGO	4958	3500	2964	2355	3110	1401	8060	2699	5343	5226	4259	5595	9264	10993	9000	12048	11180	13520	10005	8918	24339	15589
NGA	3495	6132	1830	3055	1922	3458	2753	4090	3924	6781	7403	6062	6385	9928	5318	12336	17389	15183	12095	14587	12139	12859
SUR	1145	1932	2243	2814	1808	1893	2158	4431	4227	4797	4125	13540	6628	9659	8080	10457	13718	15367	14013	11076	10113	12042
GUY	1835	2825	4216	2630	3579	3744	3346	6377	4929	6656	5831	8942	4512	7790	8463	16689	13505	7628	12964	10763	7088	7884
SLB	746	2129	1276	2201	3843	2496	3016	4826	3126	3225	3095	5253	5597	10327	9287	11672	14327	13351	12324	10458	8506	10137
BLZ	1271	5574	2998	2103	3208	1897	4123	3609	4405	4769	16295	6762	8449	9026	6600	11477	6591	4789	8745	15951	4558	4029
THA	2035	3856	4246	8034	10678	9554	11325	7816	13198	12206	8567	8092	3973	4650	2326	4806	3745	1948	2621	3573	2921	3295
GHA	1700	4438	1875	1583	3406	3654	3016	2670	3538	1648	2772	2501	5112	7456	3621	13749	7689	12289	5822	14272	10700	17970
PAN	1187	1889	1575	2478	2336	4571	3111	6855	5559	2965	4757	3115	3721	2702	2027	5905	5913	6121	6234	6593	5308	4018
ETH	1399	1569	2542	2730	2302	2562	5518	3590	3993	3455	2652	5077	6609	5632	1200	2874	7631	3798	7380	4603	4349	4005

R Code of projections towards 2030

Rpackages: Tidyverse, fpp2, ggplot2

```
library("tidyverse")

library("fpp2")

# Data load

datos <- data.frame(B = c(Deforest$World[1:31]),

              C = c(Deforest$Year[1:31]))

# Convert the data into time series

serie <- ts(datos$B, start = c(1990), frequency = 1)

# Generating the different model

modelo_autoR <- ets(serie, model = "AAN",damped=FALSE)

modelo_intermedio <- ets(serie, model = "AAN",damped=FALSE,alpha=0.9,beta=0.5)

modelo_R <- ets(serie, model = "AAN",damped=FALSE,alpha=0.9,beta=0.001)

# Model summary

summary(modelo_intermedio)

# Forecasting next years

pronostico <- forecast(modelo_intermedio, h = 10, level = c(99.9))

# Check the forecast

pronostico$mean

pronostico_df <- data.frame(date = time(pronostico$mean),

                  lower = pronostico$lower[,1],

                  upper = pronostico$upper[,1],

                  mean = pronostico$mean)

# Plotting

ggplot() +
```

```
# Rel data

  geom_line(data = datos, aes(x = C, y = B), color = "green") +

  # Forecast values

  geom_line(data = pronostico_df, aes(x = date, y = mean), color = "green", linetype = "dashed") +

  # CI at 99.9%

  geom_ribbon(data = pronostico_df, aes(x = date, ymin = lower, ymax = upper), fill = "lightblue",
alpha = 0.4) +

  # Title and names

  labs(x = "Year, y = "Value", title = "Forecast with CI at 99.9%") +

  # Plot theme

  theme((plot.title=element_text(hjust=0.5))
```

References

1. S. Sharma, N. Batra, Comparative study of single linkage, complete linkage, and ward method of agglomerative clustering. In *2019 International Conference on Machine Learning, Big Data, Cloud and Parallel Computing (COMITCon)* (pp. 568-573). IEEE (2019).
